# Leveraging pleat folds and soft compliant elements in inflatable fabric beams

**DOI:** 10.3389/frobt.2023.1267642

**Published:** 2024-01-12

**Authors:** Juan J. Huaroto, Etsel Suarez, Wangdo Kim, Emir A. Vela

**Affiliations:** ^1^ Department of Mechanical Engineering, Universidad Nacional de Ingenieria, Lima, Peru; ^2^ Department of Mechanical Engineering, Universidad de Ingenieria y Tecnologia—UTEC, Lima, Peru; ^3^ Research Center in Bioengineering, Universidad de Ingenieria y Tecnologia—UTEC, Lima, Peru

**Keywords:** soft robotics, inflatable fabric beam, pleat folds, soft compliant elements, fabric actuators

## Abstract

Inflatable fabric beams (IFBs) integrating pleat folds can generate complex motion by modifying the pleat characteristics (e.g., dimensions, orientations). However, the capability of the IFB to return to the folded configuration relies upon the elasticity of the fabrics, requiring additional pressure inputs or complementary mechanisms. Using soft compliant elements (SCEs) assembled onto pleat folds is an appealing approach to improving the IFB elasticity and providing a range of spatial configurations when pressurized. This study introduces an actuator comprising an IFB with pleat folds and SCEs. By methodologically assembling the SCEs onto the pleat folds, we constrain the IFB unfolding to achieve out-of-plane motion at 5 kPa. Besides, the proposed actuator can generate angular displacement by regulating the input pressure ( > 5 kPa). A matrix-based representation and model are proposed to analyze the actuator motion. We experimentally study the actuator’s angular displacement by modifying SCE shapes, fold dimensions, and assembly distances of SCEs. Moreover, we analyze the effects of incorporating two SCEs onto a pleat fold. Our results show that the actuator motion can be tuned by integrating SCEs with different stiffness and varying the pleat fold dimensions. In addition, we demonstrate that the integration of two SCEs onto the pleat fold permits the actuator to return to its folded configuration when depressurized. In order to demonstrate the versatility of the proposed actuator, we devise and conduct experiments showcasing the implementation of a planar serial manipulator and a soft gripper with two grasping modalities.

## 1 Introduction

The field of soft robotics has opened up new avenues to design and fabricate actuators, sensors, and mechanisms (Yasa et al., 2023). During the last decade, various actuation principles (e.g., pneumatic (Xavier et al., 2022), dielectric (Gupta et al., 2019), magnetorheological (Bastola et al., 2020), magnetic (Kim and Zhao, 2022)) have motivated the development of soft actuators for a myriad of applications, ranging from aerospace to physiatry (Huaroto et al., 2019; Yoo et al., 2019; Gollob et al., 2023; O’Neill et al., 2023; Zhang et al., 2023a). In particular, soft pneumatic actuators have been the gold standard for creating large stroke actuation. However, bulk structures made of rubber-like silicones have limited the practical integration of soft pneumatic actuators in wearable devices (Zhu et al., 2020; Yang et al., 2023). Fabric-based pneumatic actuators (FPAs) harness the properties of fabrics (e.g., light weight, flexibility, anisotropy, and softness) to enable their integration into wearable devices (Suarez et al., 2018; Connolly et al., 2019; Yoo et al., 2019; O’Neill et al., 2021), smart garments (Sanchez et al., 2021), and systems for assistive technology or human augmentation (Cappello et al., 2018a; Liang et al., 2018; Proietti et al., 2023).

The FPAs frequently use knit and woven textiles to fabricate fundamental structures such as pouches and inflatable fabric beams (IFBs) (Niiyama et al., 2015; Khin et al., 2017; Yoo et al., 2019; Nguyen and Zhang, 2020; Lee and Rodrigue, 2023). These inflatable structures are engineered to create twisting, elongating, bending, straightening, and multi-degrees-of-freedom actuators (Nguyen and Zhang, 2020; Sanchez et al., 2021. In particular, an IFB can attain a targeted motion through the incorporation of tailored sewing patterns (Yap et al., 2017a; Ge et al., 2020; Zhu et al., 2020; Suulker et al., 2022), heat-sealing hinges (Ou et al., 2016; Khin et al., 2017), anisotropic fabrics (Cappello et al., 2018b; Connolly et al., 2019), and folds (Yoo et al., 2019). While IFBs have found applications in manipulators, grippers, and assistive technology, their ability to revert to the folded configuration upon depressurization relies upon the elasticity modulus of the fabric (Nesler et al., 2018; Yoo et al., 2019). The textiles used in IFBs possess a notably higher elasticity modulus than elastomeric materials (Zhang et al., 2023b). This characteristic facilitates a swift and stable inflation response of the IFBs but limits their ability to revert to their original state. Prior research has addressed this challenge by integrating supplementary bladders along with pressure inputs (Cappello et al., 2018a), winding mechanisms (Zhang et al., 2023b), and elastomeric materials (i.e., soft compliant elements) (Natividad et al., 2017; Suarez et al., 2018).

In the realm of IFBs integrating folds, pleating techniques have motivated the development of straightening and bending actuators (Khin et al., 2017; Nesler et al., 2018; Yoo et al., 2019). A pleat fold is a fundamental origami fold consisting of valley and mountain creases. Incorporating pleat folds into an IFB serves as a method to develop deployable structures, enabling programmable motion by setting the distances between creases (Yap et al., 2017a; Yoo et al., 2019). In order to revert the IFB to its folded configuration, an appealing approach consists of integrating soft compliant elements (SCEs) onto pleat fold creases. However, this integration represents an unexplored domain in the state-of-the-art (Yoo et al., 2019). The SCEs can constrain the inherent tendency of the IFB to straighten under pressure to perform out-of-plane motion and a range of spatial configurations at varying pressure inputs. In addition, the inherent elasticity of SCEs can revert the IFB to its folded configuration upon deactivating the input pressure. Exploring the influence of geometrical characteristics of SCEs, fold dimensions, and their integration can provide insights into the IFB motion, enabling potential applications in inflatable manipulators and soft wearable devices.

In this study, we combine the benefits of using fabric and elastomeric materials to introduce an actuator that incorporates an IFB with pleat folds and SCEs. By strategically assembling the SCEs onto the pleat folds, we enable out-of-plane motion at 5 kPa and angular displacement at varying pressures ( > 5 kPa). Moreover, the elasticity of SCEs can facilitate the actuator to return to its folded configuration once depressurized. In order to validate the proposed approach, we expand the design concept, followed by a matrix-based representation and the conceptual framework to model the actuator motion. We experimentally study the actuator characteristics by evaluating different SCE shapes, fold lengths, and SCE assembly distances. Furthermore, we devise and conduct experimental proofs-of-concept to showcase the versatility of the proposed actuator.

## 2 Materials and methods

### 2.1 Design concept

**FIGURE 1:**
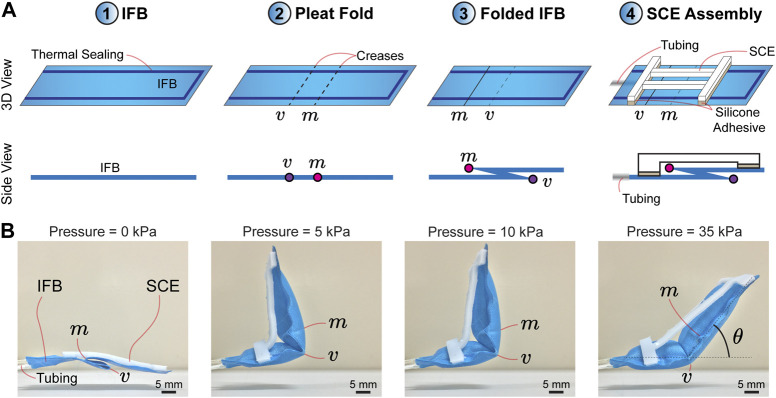
**(A)** Design concept and illustration of the proposed actuator. ① Inflatable fabric beam (IFB). ② Generation of a pleat fold comprising valley (*v*) and mountain (*m*) creases. ③ Folded IFB. ④ Assembly of a soft compliant element (SCE) onto the pleat fold. **(B)** Image frames of the actuator motion. The actuator is initially folded and deploys with respect to the crease (*m*) when powered by input pressure. The actuator achieves the maximum vertical deployment at 5 kPa. The increase in pressure leads to control of the angular displacement (*θ*) of the actuator with respect to the crease (*v*).

In order to elucidate the design concept of the proposed actuator, we consider an inflatable fabric beam (IFB) with a single pleat fold. The pleat fold comprises the following creases: valley and mountain, which are represented by the alphabet “*v*” and “*m*”, respectively (Figure 1A). The folded IFB is assembled with a soft-compliant element (SCE) of 2 mm thick, which constrains the IFB motion when pressurized. Figure 1B shows that the creases act as pivots when input pressure is activated. As a result, we divide the motion of the proposed actuator into two steps. (1) Input pressure (0–5 kPa): The actuator unfolds with respect to the crease (*m*), achieving the maximum vertical deployment at ≈ 5 kPa. (2) Input pressure ( > 5 kPa): The crease (*m*) is completely unfolded and the actuator starts to unfold with respect to the crease (*v*), performing an angular displacement (*θ*) (Figure 1A ③ and ④). The angular displacement (*θ*) can be controlled by gradually increasing the pressure (Figure 1B). Notably, the actuator tends to completely deploy (i.e., *θ* = 0) by increasing the input pressure.

### 2.2 Structural analysis of SCEs

**FIGURE 2:**
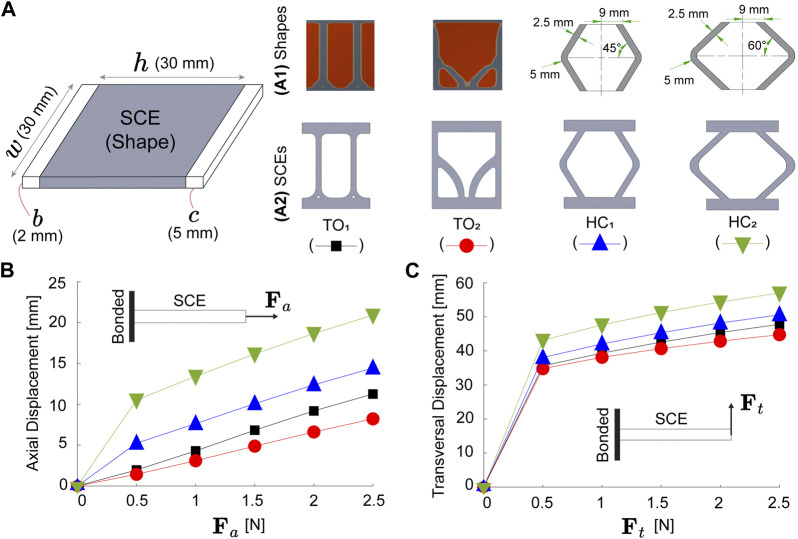
Four proposed soft compliant elements (SCEs). **(A)** Dimensions, **(A1)** shapes, and **(A2)** illustrations. The SCEs are based on topological optimization (TO_1_ and TO_2_) and honeycomb cells (HC_1_ and HC_2_). Finite element simulations of SCEs under **(B)** axial (**F**_*a*_) and **(C)** transversal (**F**_*t*_) forces.

The angular displacement (*θ*) of the actuator can be programmed by varying the stiffness of the SCE. Figure 2A shows the dimensions of the SCEs. The white parts (*w* × *c* × *b* mm^3^) are the segments used to glue the SCE on the IFB, while the gray part (*w* × *h* × *b* mm^3^) contains the shape of the SCE. In this study, we propose four geometries: Two based on topological optimization (TO_1_ and TO_2_) and two others using honeycomb unit cells (HC_1_ and HC_2_) (Figure 2A). The SCEs based on topological optimization are obtained using Ansys (version 18.0, Ansys Inc., United States). A solid rectangular plate with dimensions (30 × 30 × 2 mm^3^) is bounded by one of its sides while a force is applied on the opposite side. For obtaining the shapes TO_1_ and TO_2_, axial and transversal forces of 2 N magnitude are used. The objective function is established to maximize the deformation while keeping 25% of the plate volume. The resulting geometries are imported to SolidWorks (version 2018 SP5, SolidWorks Corp., United States) for processing. The shapes HC_1_ and HC_2_ are obtained using honeycomb unit cells characterized by angles of 45° and 60°, respectively (Figure 2A). To compare the structural stiffness of each SCE, we perform simulations in Ansys (version 18.0, Ansys Inc., United States). Figures 2B, C show the axial and transversal displacement for a range of axial (**F**_*a*_) and transversal (**F**_*t*_) forces applied at the tip of the SCE. A commercial rubber-like material (RTV-1520) is used in the simulations and, subsequently, the fabrication of the SCEs. The material is characterized by a 3-parameters (*C*_10_ = −1138 kPa, *C*_01_ = 1389 kPa, and *C*_11_ = 569 kPa) Mooney-Rivlin hyperelastic model obtained from a hyperelastic uniaxial test (ASTM D412 “Tensile Strength Properties of Rubber and Elastomers”) (Suarez et al., 2018). The simulation results reveal the higher stiffness of TO_1_ and TO_2_ compared to HC_1_ and HC_2_ for axial and transversal deformations.

### 2.3 Fabrication of components and assembly

**FIGURE 3:**
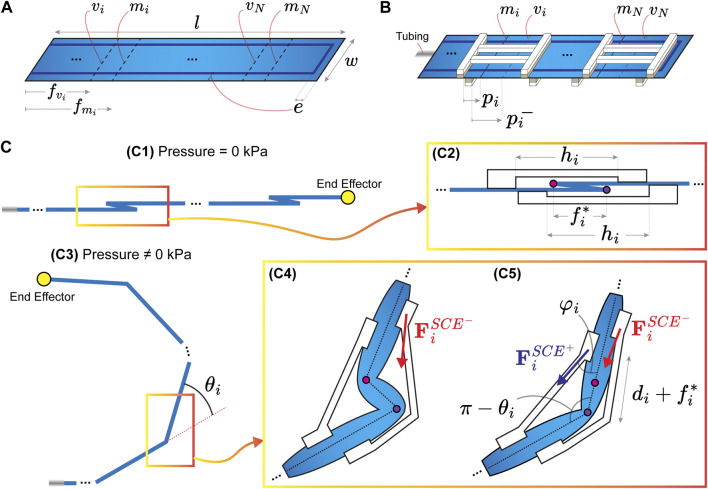
**(A)** Inflatable fabric structure (IFB) with dimensions *w* × *l* mm^2^ and *N* pleat folds. The thermosealing of the IFB has an offset (*e*) with respect to the fabric edges. The *i*th pleat fold is composed of two creases (*v*_*i*_ and *m*_*i*_), where *i* =1,2,… , *N*. The distances ( $f_{v_{i}}$ and $f_{m_{i}}$ ) correspond to each crease (*v*_*i*_ and *m*_*i*_, respectively). **(B)** Soft compliant elements (SCEs) assembled on top and underneath each pleat fold. The assembly distances are defined by (*p*_*i*_ and $p_{i}^{-}$ ), where the superscript (−) indicates that the SCE is assembled underneath the pleat fold. **(C)** Illustrations of the proposed actuator at different conditions. **(C1)** Actuator configuration at 0 kPa shows the end effector’s initial position. **(C2)** Zoom in on the *i*th pleat fold with fold distance $\left(f_{i}^{*}\right)$ assembled with two SCEs with length dimensions (*h*_*i*_). The purple and pink points indicate the creases (*v*_*i*_ and *m*_*i*_). **(C3)** Actuator configuration when powered by pressure input. **(C4)** Unfolding with respect to the crease (*m*_*i*_) showing the force $\left(F_{i}^{SCE^{-}}\right)$ due to the deformation of the SCE placed underneath the pleat fold. **(C5)** Unfolding with respect to the crease (*v*_*i*_) showing the forces $\left(F_{i}^{SCE^{-}}\;\text{and}\;F_{i}^{SCE^{+}}\right)$ relative to the SCEs.

The IFB is manufactured by cutting two sheets (*w* × *l* mm^2^) of thermoplastic polyurethane fabric (Heat Sealable 200 Denier Oxford Nylon, Rockywoods Fabrics, United States). These sheets are placed one over another and then sealed with a thermosealing machine (FS-200, HUALIAN America S.A., Mexico). The thermosealing has an offset (*e*) in millimeters with respect to the fabric edges (Figure 3A). A connecting tube is integrated with one of the ends of the IFB using instant adhesive (Loctite 401, Henkel AG and Co. KGaA, Germany). The pleat folds are manually made on the IFB using heat and pressure. The SCEs are fabricated by curing RTV-1520 silicone rubber in 3D printed (MD-6C, Shenzhen Mingda Technology Co., China) Polylactic acid (PLA) molds. The RTV-1520 components are mixed (1:1 mass ratio) and subsequently poured into the molds. Thereon, the molds containing the blend are put into a vacuum chamber and cured at room temperature for 6 h. The resulting SCEs are glued on the IFB using silicone adhesive (Sil-Poxy, SmoothOn Inc., United States).

### 2.4 Matrix-based representation

The design concept of an IFB, including a pleat fold and an SCE, provides the fundamental working principle of the proposed actuator. Aiming to provide a general and structured formulation of the IFB, pleat folds, and SCEs geometry, we present a matrix-based representation of the proposed actuator. We consider an IFB (*w* × *l* mm^2^) on which the thermosealing has an offset (*e*) with respect to the fabric edges (Figure 3A). The IFB has N∈N pleat folds; hence the *i*th pleat fold consists of two creases (*v*_*i*_ and *m*_*i*_). We begin by including the three dimensions of the IFB into a vector $\left(P={\left[w,\;l,\;e\right]}^{T}\right)$ . Thereon, the distances ( $f_{v_{i}}\in R^{+}$ and $f_{m_{i}}\in R^{+}$ ) corresponding to the creases (*v*_*i*_ and *m*_*i*_, respectively) are arranged into a matrix $\left(F\in R^{3\times N}\right)$ as follows:

$$F=\left[\begin{array}{ccc} f_{v_{1}} & \cdots & f_{v_{N}}\\ f_{m_{1}} & \cdots & f_{m_{N}}\\ f_{1}^{*} & \cdots & f_{N}^{*} \end{array}\right].$$ (1)

Using the columns of (**F**) we define the fold dimension $\left(f_{i}^{*}\right)$ of the *i*th pleat fold as: $f_{i}^{*}=f_{m_{i}}-f_{v_{i}}$ . The matrix **P** and **F** determine the geometry of an IFB with *N* pleat folds. In addition, we can compute the folded length (*l*′) of the IFB using the following expression:

$$l^{\prime }=l-2\overset{N}{\underset{i=1}{\sum }}f_{i}^{*}.$$ (2)

Following the matrix-based representation, the characteristics of SCEs are integrated into the matrix $\left(S\in R^{3\times N}\right)$ . According to the geometry of the IFB, we set a maximum of two SCEs per pleat fold (on top and underneath) (Figure 3B). The assembly distances ( $p_{i}\in R^{+}$ and $p_{i}^{-}\in R^{+}$ ) are relative to the creases (*m*_*i*_ and *v*_*i*_), respectively (Figure 3B). The subscript (−) in $p_{i}^{-}$ indicates that the distance corresponds to the SCEs underneath the pleat fold. The IFB and SCEs have the same width (*w*), and the effective length (*h*_*i*_) of each SCE is used to define the distances (*d*_*i*_ = *h*_*i*_ − *p*_*i*_ and $d_{i}^{-}=h_{i}-p_{i}^{-}$ ). The matrix (**S**) defined as follows:

$$S=\left[\begin{array}{ccc} h_{1} & \cdots & h_{N}\\ d_{1}\;\left(□\right) & \cdots & d_{N}\;\left(□\right)\\ d_{1}^{-}\;\left(□\right) & \cdots & d_{N}^{-}\;\left(□\right) \end{array}\right],$$ (3)

the symbol (□) indicates the type of the SCE (TO_1_, TO_2_, HC_1_, and HC_2_). The proposed actuator integrates the matrices (**P**, **F**, and **S**), which contain the main parameters to describe the actuator mathematically. We define the matrix $\left(A\in R^{3\times \left(2N+1\right)}\right)$ to represent the actuator and is defined as follows:

$$A=\left[\begin{array}{ccc} P & F & S \end{array}\right].$$ (4)

### 2.5 Modeling

In order to complement the matrix-based representation of the actuator, we present a forward kinematics model based on the associated energy of the folds $\left(W^{f}\right)$ and the deformation energy per volume unit of the SCEs $\left(W^{\mathrm{SCE}}\right)$ . The total energy of the folds is computed as the sum of energy associated with each crease (*v*_*i*_) existing along the IFB fold. In order to compute *W* ^*f*^, we utilize the following equation (Nesler et al., 2018):

$$W^{f}=\overset{N}{\underset{i=1}{\sum }}T_{i}\;\left(\pi -\theta_{i}\right),$$ (5)

Where $T_{i}\in R$ and $\theta_{i}\in R$ are the torque and the angular displacement with respect to the crease (*v*_*i*_). The energy associated with the SCEs is the deformation energy per unit of volume and is defined using a 3-parameter Mooney-Rivlin hyperelastic model (Suarez et al., 2018). The deformation energy per volume unit of SCE associated with the *i*th pleat fold can be as follows:

$$\begin{array}{ll} W_{i}^{SCE^{+,-}} & =\;\left[C_{10}\;\left(I_{1}-3\right)+C_{01}\;\left(I_{2}-3\right)+C_{11}\;\left(I_{1}-3\right)\;\right.\\ & \;\;\;{\left.\left(I_{2}-3\right)\right]}_{i}^{+,-}, \end{array}$$ (6)

Where the superscripts (+, −) indicate the position (top and underneath) of the SCE. Besides, *I*_1_, *I*_2_, and *I*_3_ are the principal invariants of the Cauchy - Green tensor. Each invariant is defined using the principal stretch ratios *λ*_1_, *λ*_2_, and *λ*_3_ (with 1, 2, and 3 as the principal axes). The following equations define the principal invariants:

$$I_{1}=\lambda_{1}^{2}+\lambda_{2}^{2}+\lambda_{3}^{2}$$ (7)

$$I_{2}=\lambda_{1}^{2}\;\lambda_{2}^{2}+\lambda_{2}^{2}\;\lambda_{3}^{2}+\lambda_{3}^{2}\;\lambda_{1}^{2}$$ (8)

$$I_{3}=\lambda_{1}^{2}\;\lambda_{2}^{2}\;\lambda_{3}^{2}=1.$$ (9)

Using Eq. (6), the equation to compute the total deformation energy per unit of volume $\left(W^{SCE^{+,-}}\right)$ is as follows:

$$W^{SCE^{+,-}}=\overset{N}{\underset{i=1}{\sum }}\left(W_{i}^{SCE^{+}}+W_{i}^{SCE^{-}}\right).$$ (10)

In order to simplify the model, we assume only the axial deformation ratio in the SCEs (see Figure 3(C5)). Therefore, we approximate *λ*_2_ ≈ 1 (since the SCEs do not bend significantly about its axial axis), hence *λ*_1_ = *λ* and $\lambda_{3}=\frac{1}{\lambda }$ (using Eq. 9). The mechanical stress associated with each SCE is calculated using two Eq. 1 Using the ratio between axial force and SCE cross-section 2 Using the hyperelasticity theory with *λ*_2_ ≈ 1. Summarizing both equations, we obtain the following:

$$\sigma_{i}^{+,-}=\frac{F_{i}^{SCE^{+,-}}}{dS_{i}}=\frac{\partial \;W_{i}^{SCE^{+,-}}}{\partial \;\lambda_{i}}\;\lambda_{i}.$$ (11)

Where $S_{i}\in R^{+}$ is the SCE cross-section and $F_{i}^{SCE^{+,-}}\in R^{2}$ are the forces associated with the SCEs according to their relative position with the *i*th pleat fold. At the equilibrium, the forces $F_{i}^{SCE^{+,-}}$ are used to calculate the numerical value of the torques (*T*_*i*_) associated with the *i*th pleat fold. Thus, by using the illustration depicted in Figure 3(C5), we formulate the following expression:

$$T_{i}=\left(d_{i}+f_{i}^{*}\right)\sin\left(\varphi_{i}\right)\;F_{i}^{SCE^{+}}.$$ (12)

It is worth noting that the torque caused by force $\left(F_{i}^{SCE^{-}}\right)$ is neglected because its orientation is approximated to intersect the crease (*m*_*i*_). Using the illustration (see Figure 3(C5)) and trigonometric relationships, the value of *φ*_*i*_ is computed as follows:

$$\varphi_{i}=\arcsin\left(\left(p_{i}+f_{i}^{*}\right)\;\frac{\sin\left(\theta_{i}\right)}{\epsilon_{i}^{*}}\right),$$ (13)

Where $\epsilon_{i}^{*}$ is the deformation length of the SCE assembled on top of the pleat fold. The actuator’s total energy (*W*_*T*_) is equal to the pneumatic energy (*W*_*p*_ = *PV*), where *P* is the internal pressure and *V* is the volume of the actuator. At the equilibrium, the energies $\left(W^{f}\;\text{and}\;W^{\mathrm{SCE}}\right)$ are equal. This way, we obtain the following equations: *W*_*T*_ = *P dV* = 2 *W* ^*f*^ = 2 *W* ^*SCE*^. In order to calculate the total volume of the IFB, we utilize the following equation, taking into account the buckling angle at each crease (*m*_*i*_) (Nesler et al., 2018):

$$V=\pi \;r^{2}\left(l-2r\overset{N}{\underset{i=1}{\sum }}\left(\tan\left(\frac{\theta_{i}}{2}\right)\right)\right),$$ (14)

Where *r* is the approximated cross-sectional radius of the IFB when pressurized. By differentiating the total energy with respect to *θ*_*i*_, we obtain the following equation:

$$\frac{\partial W_{T}}{\partial \theta_{i}}=\frac{2\;\partial W^{f}}{\partial \theta_{i}}=\frac{2\;P\partial V}{\partial \theta_{i}}.$$ (15)

Solving 15 by substituting Eqs (6)–(14) permits to obtain the angles (*θ*_*i*_) at a given pressure *P*. The resulting angles are used to determine the forward kinematic model of the proposed actuator. Figure 3(C1) and (C3) are the initial and deployed positions of the IFB, respectively. Using 2, we obtain the initial position $\left(P_{\text{initial}}={\left[l^{\prime },\;0\right]}^{T}\right)$ of the actuator end effector. Following the illustration depicted in Figure 3(C3), we compute the end-effector’s final position $\left(P_{\text{final}}\in R^{2}\right)$ using Euler notation as follows:

$$P_{\text{final}}=\overset{N}{\underset{k=0}{\sum }}\left(\left(f_{v_{k+1}}-f_{v_{k}}\right)\overset{k}{\underset{s=0}{\prod }}\left(e^{j\;\theta_{s}}\right)\right),$$ (16)

where $j=\sqrt{-1}$ , $f_{v_{0}}=0$ , $f_{v_{N+1}}=l$ , and *θ*_0_ = 0.

### 2.6 Instrumentation

**FIGURE 4:**
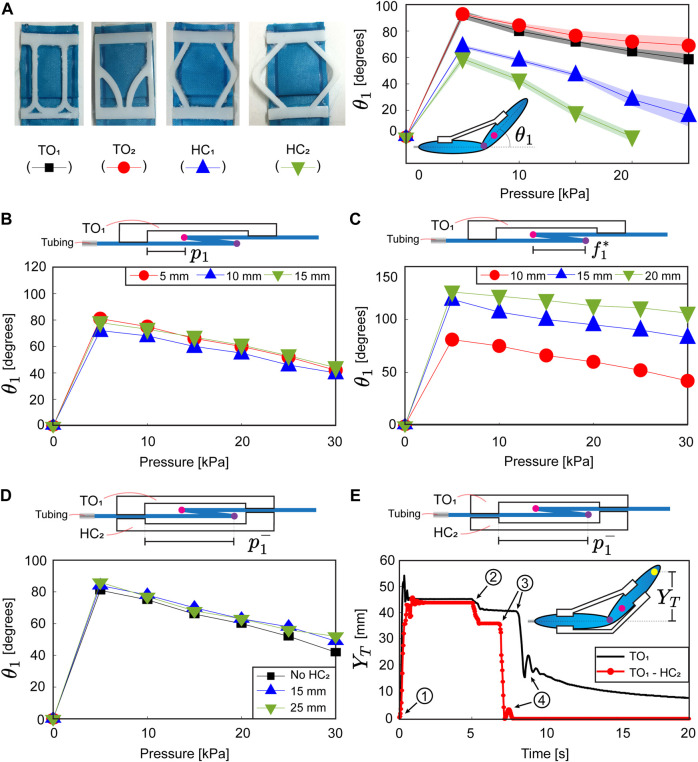
**(A)** Four actuators using the proposed soft compliant elements (SCEs) and angular displacement (*θ*_1_) for a set of pressure inputs. Angular displacement at varying **(B)** assembly distances (*p*_1_) and **(C)** fold lengths $\left(f_{1}^{*}\right)$ . **(D)** Effect of using an additional SCE assembled underneath the pleat fold. **(E)** Dynamic response of an actuator, using an additional SCE assembled underneath the pleat fold. ① the input pressure is activated at 15 kPa. ② the input pressure is deactivated. ③ the actuator deflates and starts to fold with respect to the crease (*m*). ④ the actuator tends to return to its initial position. The purple and pink points indicate the creases (*v* and *m*, respectively).

The actuator is powered using a pressurized air source controlled by a pressure regulator (P31R Series, Parker Hannifin Corp., United States). We measure the pressure supplied to the actuator using a sensor (ASDX 100PGAA5, Honeywell International Inc., United States). The pressure sensor is connected to an analog-to-digital converter (Arduino Uno, Arduino, Italy), which communicates to a computer through a USB 3.0 interface (10 kHz sampling frequency). A camera (Hero 6, GoPro, United States) records images and videos of the actuator in response to applied internal pressure. For static experiments, the images are acquired in the field of view 2040 × 2040 pixels. The experimental results for the angular displacement are averaged over 3 datasets. A custom script of MATLAB (version 2017, MathWorks, United States) is employed to obtain the angular displacement (*θ*_1_) (Figures 4A–D). The camera records videos in the field of view 1920 × 1080 pixels for dynamic experiments using a maximum frame rate of 120 frames per second (fps). The vertical displacement (*Y*_*T*_) achieved by the actuator is obtained through a custom script of MATLAB (Figure 4E).

**FIGURE 5:**
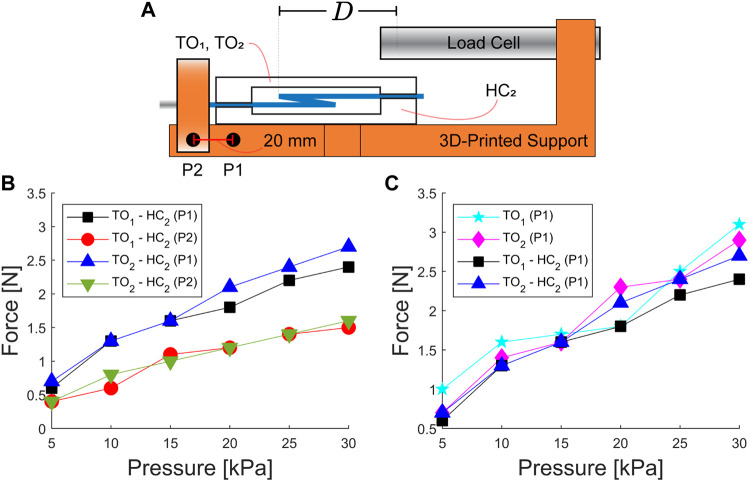
Unfolding force characterization. **(A)** Illustration of the experimental setup utilized to acquire the force exerted by the actuators. The points (P1 and P2) are used to vary the relative distance (*D*) between the actuator crease (*m*) and the load cell tip. **(B)** Unfolding force obtained for two types of actuators (TO_1_–HC_2_ and TO_2_–HC_2_) using the two positions (P1 and P2). **(C)** Folding force of actuators using a single SCE (TO_1_ and TO_2_) and two SCEs (TO_1_–HC_2_ and TO_2_–HC_2_) at the position (P1).

The force characterization is carried out using a load cell (CZL635, Phidgets Inc., Canada) fixed on a 3D-printed structure (Figure 5A). The actuator is located and secured on the 3D-printed structure for analysis. The pressure pumped through the actuator is measured using the setup previously described to analyze the actuator motion. Aiming to acquire the unfolding forces produced by the actuator, we set a gap (≈10 mm) between the load cell and IFB (Figure 5A). The analog signals acquired from the load cell are transmitted to a Wheatstone bridge amplifier (Bridge 4-Input, Phidgets Inc., Canada), which is connected to a computer using a USB 3.0 interface (sampling frequency of 125 Hz). The data acquired are processed, averaged over 3 datasets, and displayed in a Python interface (version 2.7.1).

## 3 Results

In this section, we analyze the actuator motion and unfolding force at varying pressure inputs. Besides, we test the assembly of two SCEs onto the pleat fold to explore the actuator’s capability to return to its folded configuration when depressurized. Two experimental proofs-of-concept are devised and conducted to validate the capabilities of the proposed actuator. (1) A planar serial manipulator. (2) A gripper with two grasping modalities.

### 3.1 Angular displacement

We commence our analysis by experimentally obtaining the angular displacement of an actuator using the proposed SCEs (TO_1_, TO_2_, HC_1_, HC_2_) (Figure 4A). We fabricate an IBF (**P** = [30, 100, 5]^*T*^) with a single pleat fold ( $f_{v_{1}}$ = 55 mm and $f_{1}^{*}$ = 10 mm). Each SCE is glued on top of the pleat fold according to the assembly distance (*p*_1_ = 5 mm). Figure 4A shows the angular displacement (*θ*_1_) for a set of pressures (0–25 kPa). The actuator’s maximum vertical deployment is achieved at approximately 5 kPa. For pressures ( > 5 kPa), the SCE starts to deform axially, directly impacting the angular displacement of the actuator (Figure 3(C5)). Actuators integrating SCEs characterized by relatively lower stiffness (HC_1_, HC_2_) exhibit a greater tendency to completely deploy (*θ*_1_ → 0) compared to those employing SCEs with higher stiffness (TO_1_, TO_2_). Understanding the influence of the SCE stiffness on the actuator design permits tuning the angular displacement at varying pressure inputs. Following our analysis, we vary the SCE assembly distance (*p*_1_ = 5, 10, 15 mm), which is measured with respect to the crease (*m*). In order to prevent the actuator from fully deploying, we utilize the SCE with higher stiffness (TO_1_). Figure 4B shows that the maximum vertical deployment of the actuators is achieved at 5 kPa. For higher pressures ( > 5 kPa), we observe that the assembly distance does not noticeably influence the angular displacement (Figure 4B). We explain the previous result using the illustration depicted in Figure 3(C5). The force $\left(F_{1}^{SCE^{+}}\right)$ , orientation angle (*φ*_1_), and lever arm $\left(d_{1}+f_{1}^{*}=h_{1}-p_{1}+f_{1}^{*}\right)$ are used to compute the torque attributed to the crease (*v*). Considering a set input pressure ( > 5 kPa), an increase in *p*_1_ reduces the lever arm magnitude while simultaneously increasing *φ*_1_. This increase in *φ*_1_ approximately compensates the reduced lever arm to attain the static equilibrium (Eq. 12). We experimentally demonstrate that for a set input pressure, the equilibrium is achieved at approximately the same angular displacement independent of the value of *p*_1_. Continuing our analysis, we test the angular displacement by varying the actuator fold length ( $f_{1}^{*}$ = 10, 15, 20 mm). Our results show that the fold length can modify the capability of the actuator to generate angular displacement (Figure 4C). It is worth noting that the SCE effective length (*h*_1_) stretches until a limit (*h*_max_) imposed by the condition of total deployment $\left(h_{1}^{\max}=h_{1}+2f_{1}^{*}\right)$ (Figure 3(C5)). Hence, $h_{1}^{\max}$ increases with the fold length, contributing to reducing the capability of the actuator to produce angular displacement (Figure 4C).

### 3.2 Actuator with two SCEs

The analysis of the angular displacement using a single SCE demonstrates that the actuator motion can be programmed by selecting the axial stiffness of the SCE and varying the fold length. Here, we study the effect of using two SCEs (glued on top and underneath the pleat fold). For the analysis, we use the previously fabricated IFB (**P** = [30, 100, 5]^*T*^) with a single pleat fold ( $f_{v_{1}}$ = 55 mm and $f_{1}^{*}$ = 10 mm) and (TO_1_) assembled on top of the pleat fold (*p*_1_ = 5 mm). The assembly distance of the second SCE $\left(p_{1}^{-}\right)$ is measured with respect to the crease (*v*). In order to understand the effect of using an additional SCE, we use the illustration depicted in Figure 3(C4). The SCE placed underneath the pleat fold exerts a force $\left(F_{1}^{SCE^{-}}\right)$ when the actuator unfolds with respect to the crease (*m*) (input pressure ≤ 5 kPa). To facilitate the unfolding process, we use HC_2_, which features the lowest stiffness among the proposed SCEs. Figure 4D shows the angular displacement for the distances ( $p_{1}^{-}$ = 15, 25 mm) compared to an actuator using a single SCE (TO_1_). Our results show that the additional SCE and the assembly distance $\left(p_{1}^{-}\right)$ do not significantly influence the angular displacement for a set pressure. In line with the illustration depicted in Figure 3(C5), the torques with respect to the crease (*v*) are generated from the SCE forces. We consider that the force $\left(F_{1}^{SCE^{-}}\right)$ attributed to HC_2_ approximately intersects the crease (*v*). Hence, the resulting torque can be neglected when compared to the torque exerted by the force $\left(F_{1}^{SCE^{+}}\right)$ attributed toTO_1_ (Figure 3(C5)).

The previous result demonstrates that the additional SCE does not contribute to the angular displacement of the actuator at the static equilibrium. Aiming to explore the dynamic effects of the additional SCE, we experimentally study the actuator response for a pressure pulse of 15 kPa. Two actuators (one with a single SCE and the other with two SCE) are fabricated to compare their dynamic response. To this end, we use the following characteristics: IFB dimensions (**P** = [30, 100, 5]^*T*^), fold length ( $f_{1}^{*}$ = 10 mm), and assembly distances (*p*_1_ = 5 mm and $p_{1}^{-}$ = 15 mm). The actuators’ responses are experimentally obtained by acquiring a time series of the vertical displacement (*Y*_*T*_) (Figure 4E). The pressure input (15 kPa) is activated and deactivated at t = 0 s and t = 5 s, respectively. Our results show that the additional SCE increases the actuator response time from 0.8 s to 1.9 s and acts as a damper to reduce the overshoot without substantially comprising the steady displacement (*Y*_*T*_ ≈ 45 mm) (details in Supplementary Figure S1). The force $\left(F_{1}^{SCE^{-}}\right)$ exerted by the additional SCE (depicted in Figure 3(C4)) facilitates the actuator’s return to the folded configuration (*Y*_*T*_ = 0 mm) within 2.8 s upon depressurization. In contrast, the actuator equipped with a single SCE does not completely revert to the folded configuration after 15 s of pressure deactivation.

### 3.3 Force

Understanding the static/dynamic behavior of the proposed actuator permits tuning the angular displacement and facilitating the actuator to return to the folded configuration. Here, we expand our study to analyze the unfolding force generated at varying input pressure. Previous analysis using the illustration in Figure 3(C4) has demonstrated that the force $\left(F_{1}^{SCE^{-}}\right)$ of the additional SCE mainly determines the unfolding capability with respect to the crease (*m*). Following this approach, we use the SCE with the lowest stiffness (HC_2_) for analyzing the unfolding force. For experiments, we utilize two actuators (TO_1_–HC_2_ and TO_2_–HC_2_) using the following characteristics: IFB dimensions (**P** = [30, 100, 5]^*T*^), fold length ( $f_{1}^{\;*}$ = 10 mm), and assembly distances (*p*_1_ = 5 mm and $p_{1}^{-}$ = 15 mm). A load cell acquires the unfolding force as the actuator is pressurized (Figure 5A). The actuators are fixed on a 3D-printed structure, such that the distance (*D*) from crease (*m*) to the load cell is set according to the positions (P1 and P2) (Figure 5A). Figure 5B shows the acquired forces for the actuators using a set of input pressures (5–30 kPa) and the positions (P1 and P2). We observe that both actuators exert approximately equal forces with a maximum magnitude of 2.5 N at 30 kPa. However, by modifying the positions (from P1 to P2), there is a maximum decrement of 66.6% in the acquired force due to localized buckling observed in the IFB. The actuators with a total weight of 3.4 g (≈0.034 N) can exert a force of approximately 2.5 N. Actuators using a single SCE (TO_1_ or TO_2_) are experimentally tested, showing a slight increment in force with respect to the actuators equipped with two SCEs (Figure 5C).

### 3.4 Experimental proof-of-concept

**FIGURE 6:**
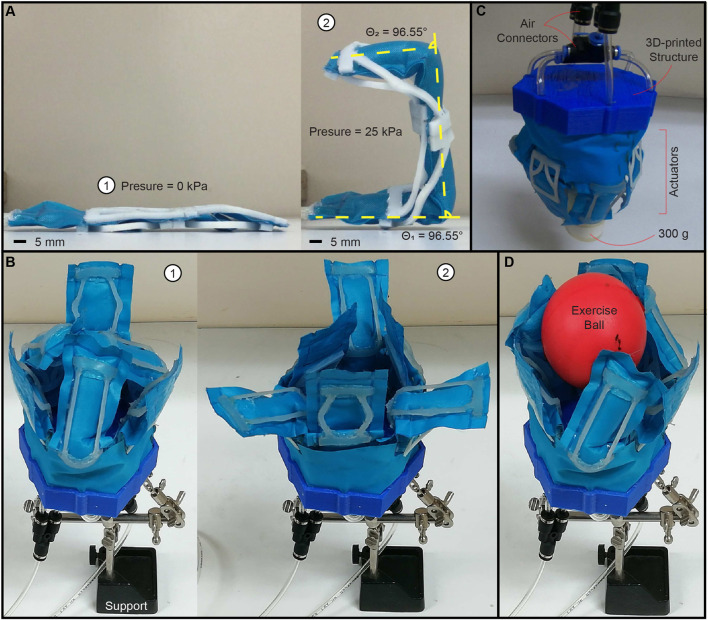
Experimental proof-of-concept. **(A)** Planar serial manipulator using an Inflatable fabric beam (IFB) with two joints (i.e., pleat folds) equipped with four soft compliant elements (SCEs). ① Folded configuration.② deployed condition. The dashed and yellow lines represent the manipulator angles obtained numerically (details in Supplementary Figure S1). **(B)** Gripper composed of six actuators. ① grasping modality 1. ② grasping modality 2. **(C)** Gripper used for lifting an object of 300 g. **(D)** Gripper grasping a hand exercise ball (using modality 1). *Please refer to the accompanying video for the demonstration of all experimental proofs-of-concept.*

In order to demonstrate the versatility of the proposed actuator, we propose two demonstrations. (1) A planar serial manipulator. (2) A gripper with two grasping modalities. For the first demonstration, we develop two manipulators, each comprising of an IFB equipped with two and three joints (i.e., pleat folds), respectively. On each joint, two SCEs are integrated following the matrix-based representations: $P={\left[30,\;150,\;5\right]}^{T},$ $F\;=\;\left[\begin{array}{cc} 55 & 110\\ 65 & 120\\ 10 & 10 \end{array}\right]\;,\;S=\;\left[\begin{array}{cc} 30 & 30\\ 25\;\left({\text{TO}}_{2}\right) & 25\;\left({\text{TO}}_{2}\right)\\ 15\;\left({\text{HC}}_{2}\right) & 15\;\left({\text{HC}}_{2}\right) \end{array}\right]$ and $P\;={\left[30,\;200,\;5\right]}^{T},$ $F=\left[\begin{array}{ccc} 55 & 110 & 165\\ 65 & 120 & 175\\ 10 & 10 & 10 \end{array}\right],\;S=\left[\begin{array}{ccc} 30 & 30 & 30\\ 25\;\left({\text{TO}}_{2}\right) & 25\;\left({\text{TO}}_{2}\right) & 25\;\left({\text{TO}}_{2}\right)\\ 15\;\left({\text{HC}}_{2}\right) & 15\;\left({\text{HC}}_{2}\right) & 15\;\left({\text{HC}}_{2}\right) \end{array}\right]$ . Figure 6A shows the serial manipulator with two joints at the folded configuration (①) and deployed condition (②). The manipulator with three joints is powered using 25 kPa to complete three actuation cycles (S1 Video, Supplementary Material: 01:31–01:58 s). We demonstrate that the proposed actuator can be extended for multiple pleat folds, enabling out-of-plane motion capabilities. For the second demonstration, we design a gripper composed of six actuators with the following characteristics: $P={\left[40,\;100,\;7\right]}^{T},$ $\;F=\left[\begin{array}{c} 25\\ 35\\ 10 \end{array}\right],\;S=\left[\begin{array}{c} 30\\ 25\;\left({\text{TO}}_{2}\right)\\ 30\;\left({\text{HC}}_{1}\right) \end{array}\right]$ . The actuators are interspersed along a fabric ring assembled on a 3D-printed structure, permitting two grasping modalities (Figure 6B). Figure 6D shows the gripper using one modality to grasp a hand exercise ball. The experimental validation of the two-modality gripper is addressed by performing pick and place operations of three objects with different shapes (S1 Video, Supplementary Material: 02:59–03:46 s). The gripper weighting 21g (excluding the 3D-printed support) is successfully tested for lifting a solid element of 300 g (Figure 6C), resulting in a payload-to-weight ratio of 1428%. We emphasize the distinctive attributes of the proposed gripper by leveraging the properties of the Inflatable Foldable Structure (IFB) with pleat folds and Soft Compliant Elements (SCEs) in comparison to other grippers and gloves within the current state-of-the-art (Table 1).

**TABLE 1:** Comparison of developed gripper with existing fabric-based pneumatic grippers (Gr) and gloves (Gl).

References	Type	Pleat folds	Weight [g]	Payload [g]	Payload-to-weight ratio [%]	Pressure [kPa]
Nishioka et al. (2012)	Gr	No	13.2	500	3787	20
Yap et al. (2017a)	Gl	Yes	99	800	808	70
Yap et al. (2017b)	Gl	No	180	454	252	120
Khin et al. (2017)	Gr	No	140	2400	1714	30
Nguyen et al. (2019)	Gr	No	-	2000	-	200
Zhang et al. (2023b)	Gr	No	12	7000	58333	50
**This study**	**Gr**	**Yes**	**21**	**300**	**1428**	**25**

Bold font represents to highligh the current study.

## 4 Discussion

In summary, we introduce an actuator comprising an inflatable fabric beam (IFB) with pleat folds and soft compliant elements (SCEs). Integrating SCEs with pleat folds permits a range of spatial configurations. Besides, it contributes to facilitating the return of the IFB to the folded state. The proposed actuator has a maximum thickness of 4 mm and achieves the maximum vertical deployment at 5 kPa. The increase in pressure ( > 5 kPa) allows the actuator to describe an angular displacement with respect to the valley crease. In order to analyze the fold parameters and assembly distances of SCEs, we introduce a matrix-based representation followed by a model to provide insights into the actuator motion. We experimentally demonstrate that the SCE stiffness and fold length can modify the actuator’s capability to produce angular displacement. In addition, we find that integrating an additional SCE (underneath the pleat fold) permits the actuator to return to the folded configuration once the input pressure is deactivated. The unfolding force exerted by the actuator (3.4 g) is experimentally obtained, resulting in 2.5 N (at 30 kPa). The validation of the actuator functionalities to develop planar serial manipulators and a gripper with two grasping modalities motivates the integration into functional garments and soft wearable devices. Future work includes using complex inflatable fabric structures, origami-based folds, novel SCE shapes, an extended formulation of the matrix-based representation, and a comprehensive analysis of the actuator dynamics for various actuation cycles.

## Data Availability

The original contributions presented in the study are included in the article/Supplementary Material, further inquiries can be directed to the corresponding authors.
