# Supplementary material for: Leveraging pleat folds and soft compliant elements in inflatable fabric beams
Source: Front Robot AI. 2024 Jan 12;10:1267642. doi: 10.3389/frobt.2023.1267642 (PMC10822686; doi:10.3389/frobt.2023.1267642)
Supplement: Supplementary file 1 [file DataSheet1.pdf]

Supplementary Information

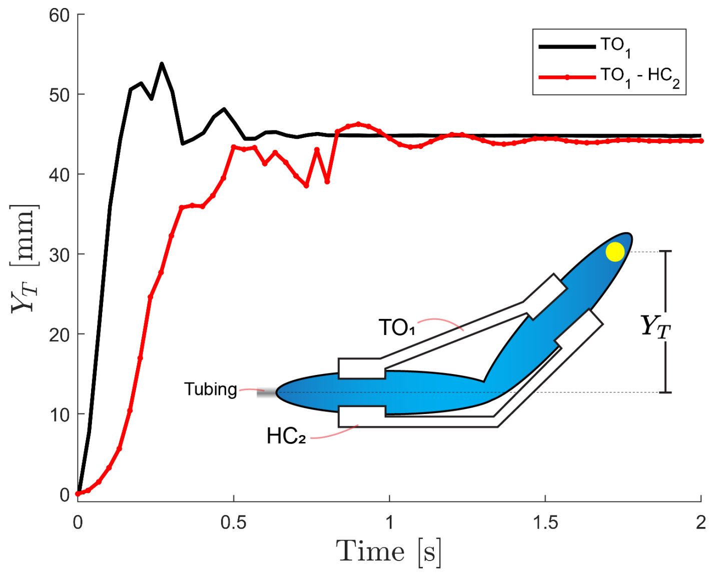

Figure S1. Dynamic response of two actuators integrating a single (TO_1) and two soft-compliant elements (TO_1–HC_2). The actuators TO_1 and TO_1–HC_2 show a response time of 0.8 s and 1.9 s, respectively. In addition, the steady-state error on the vertical displacement (Y_T) between actuators is 1.41 %.

1 MODEL VALIDATION

We validate the proposed model using the experimental results obtained from the planar serial manipulator with two joints (i.e., pleat folds). We define the associated energy of the folds (W^f) as follows:

$$W^f = T_1\,(\pi - \theta_1) + T_2\,(\pi - \theta_2). \tag{S1}$$

The torques (T_1 and T_2) are computed using Equations (12) and (13) as follows:

$$T_1 = (d_1 + f_1^*)(p_1 + f_1^*)\,\frac{\sin(\theta_1)}{\epsilon_1^*}\,F_1^{SCE^+}, \tag{S2}$$

$$T_2 = (d_2 + f_2^*)(p_2 + f_2^*)\,\frac{\sin(\theta_2)}{\epsilon_2^*}\,F_2^{SCE^+}. \tag{S3}$$

The assembly parameters of the SCEs with respect to the pleat folds are constant according to the matrix-based representation of the manipulator: $\mathbf{P} = [w = 30,\, l = 150,\, e = 5]^T$mm, $\mathbf{F} = \begin{bmatrix} f_{v_1} = 55 & f_{v_1} = 110 \\ f_{m_1} = 65 & f_{m_2} = 120 \\ f_1^* = 10 & f_2^* = 10 \end{bmatrix}$ mm, $\mathbf{S} = \begin{bmatrix} h_1 = 30 & h_2 = 30 \\ d_1 = 25\,(\mathrm{TO_2}) & d_2 = 25\,(\mathrm{TO_2}) \\ d_1^- = 15\,(\mathrm{HC_2}) & d_2^- = 15\,(\mathrm{HC_2}) \end{bmatrix}$ mm. In addition, the ratio ($\frac{F_i^{SCE^+}}{\epsilon_i^*}$) can be approximated as the SCE axial stiffness (K_i). The axial stiffness of TO_2 is linearly

approximated ($k = k_1 = k_2 = 306.26$ N/m) from the results shown in Figure 2B. Using equation (14), we compute the volume of the inflatable fabric beam (IFB):

$$V = \pi r^2 \left(l - 2r \left(\tan\left(\frac{\theta_1}{2}\right) + \tan\left(\frac{\theta_2}{2}\right) \right) \right). \tag{S4}$$

The effective cross-sectional radius (r) of the IFB is approximated using the following equation:

$$r = \frac{(w - 2\,e)}{\pi}, \tag{S5}$$

Using equation S1, we obtain the following mathematical expressions for $i = 1, 2$:

$$\frac{\partial W^f}{\partial \theta_i} = k\,(d_i + f_i^*)(p_i + f_i^*)\cos(\theta_i)\,\theta_i + T_i. \tag{S6}$$

In addition, using equation S4, the derivatives of the volume with respect to the angles (θ_1 and θ_2) are as follows:

$$\frac{\partial V}{\partial \theta_i} = -\pi\, r^3\, \sec^3(\theta_i). \tag{S7}$$

We introduce a correction factor (C_f) in Equation (15) to obtain the following relationship: $\frac{\partial W^f}{\partial \theta_i} = C_f \frac{P \partial V}{\partial \theta_i}$, where P is the input pressure (25 kPa). We replace S6 and S7 into equation (15) to numerically obtain the angular displacement corresponding to each fold. The obtained angles ($\theta_1 = \theta_2 = 96.55$ degrees) are numerically identical since the SCEs and assembly parameters remain consistent for each pleat fold.
